# Complete Spontaneous Regression of Colorectal Cancer: A Report of Two Cases

**DOI:** 10.7759/cureus.39128

**Published:** 2023-05-17

**Authors:** Paul W Shuttleworth, Sana Ullah, Michael Scott, Shariq Sabri, Mamoon Solkar

**Affiliations:** 1 General Surgery, Tameside General Hospital, Manchester, GBR; 2 General and Colorectal Surgery, Tameside General Hospital, Manchester, GBR; 3 Histopathology, Manchester University NHS Foundation Trust, Manchester, GBR; 4 Surgery, Tameside General Hospital, Manchester, GBR

**Keywords:** histology, cancer immunotherapy, spontaneous regression of cancer, cancer colon, colorectal cancer

## Abstract

Spontaneous regression of cancer is rare, and rarer still in colorectal cancer. We present a report of two cases of spontaneous regression of histologically proven proximal colonic cancers described in detail, alongside endoscopic, histological, and radiological images. We discussed the potential mechanisms by reviewing previous literature.

## Introduction

Spontaneous regression of cancer is a rare, but well-recognized phenomenon with the term coined by Cole and Everson in their 1956 review [1]. It represents the complete or partial disappearance of a primary and/or disseminated lesion, of a histologically proven malignant lesion, without disease-specific treatment, or when treatment would be deemed inadequate. This rare entity has long been recognized in almost all cancer types but is relatively more common in some forms of cancer, particularly malignant melanoma, leukemia, and Merkel cell carcinoma [2]. There have been several reviews published with Challis and Stam finding that around 20 cases were published each year up to 1987 [3]. There are different estimates of the incidence but it is thought to occur in between one in 60,000 and one in 100,000 cases. Abdelrazeq carried out a comprehensive review in 2007, specifically for colorectal cancer. He found that less than 2% of published cases of the spontaneous regression of cancer were related to colorectal cancer [4]. Colorectal cancer is not a cancer that is relatively well recognized to undergo spontaneous regression, and a recent review of gastrointestinal malignancies undergoing spontaneous regression identified only 60 published cases [5]. Papac discussed the historical background of the spontaneous regression of cancer of all types in his 1996 review [6]. He found very few published cases related to colorectal cancers, and that most case reports did not discuss mechanisms, merely publishing the occurrence.

We present a short report of two cases of spontaneous regression of colorectal cancer identified within our unit and describe their cases in detail.

## Case presentation

Case 1

A 78-year-old female was referred to the surgical outpatient department in June 2018 on an HSC205 urgent cancer referral basis with eight weeks of diarrhea, opening her bowels up to eight times a day. She lost 13 kg weight over the last six months. There was no history of bleeding per rectum (PR) or mucous. She had mild iron deficient anemia with hemoglobin 114 g/L, MCV 76 fL, and ferritin 11 ng/mL.

She had a medical history of bladder cancer (G1T3 transitional cell carcinoma {TCC} in 2013 treated with 12 months of BCG), total abdominal hysterectomy and bilateral salpingo-oophorectomy for endometriosis, congestive cardiac failure, hypertension, diet-controlled type 2 diabetes, chronic kidney disease (CKD) stage 3, and gout. She was an ex-smoker, with no significant personal or family history of bowel disease. Her medications include ramipril, bisoprolol, omeprazole, and allopurinol.

**Figure 1:**
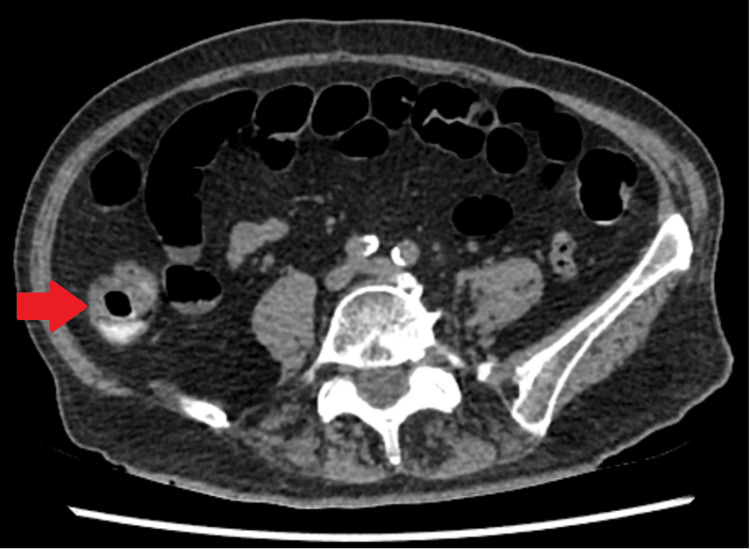
CTVC demonstrating ascending colonic thickening. CTVC: computed tomography virtual colonoscopy

A colonoscopy was ordered which was performed urgently, on a two-week basis. Unfortunately, it was incomplete due to tortuosity and narrowing of the sigmoid colon secondary to diverticular disease and patient discomfort. A CT virtual colonoscopy was arranged which showed a 2.2 cm eccentric segment of thickened mid-ascending colon suspicious of a neoplastic lesion (Figure 1). The CT scan was compared with CT scans performed in 2013 and 2016, performed due to her bladder cancer; these did not show the lesion, although they were not dedicated colonic studies.

**Figure 2:**
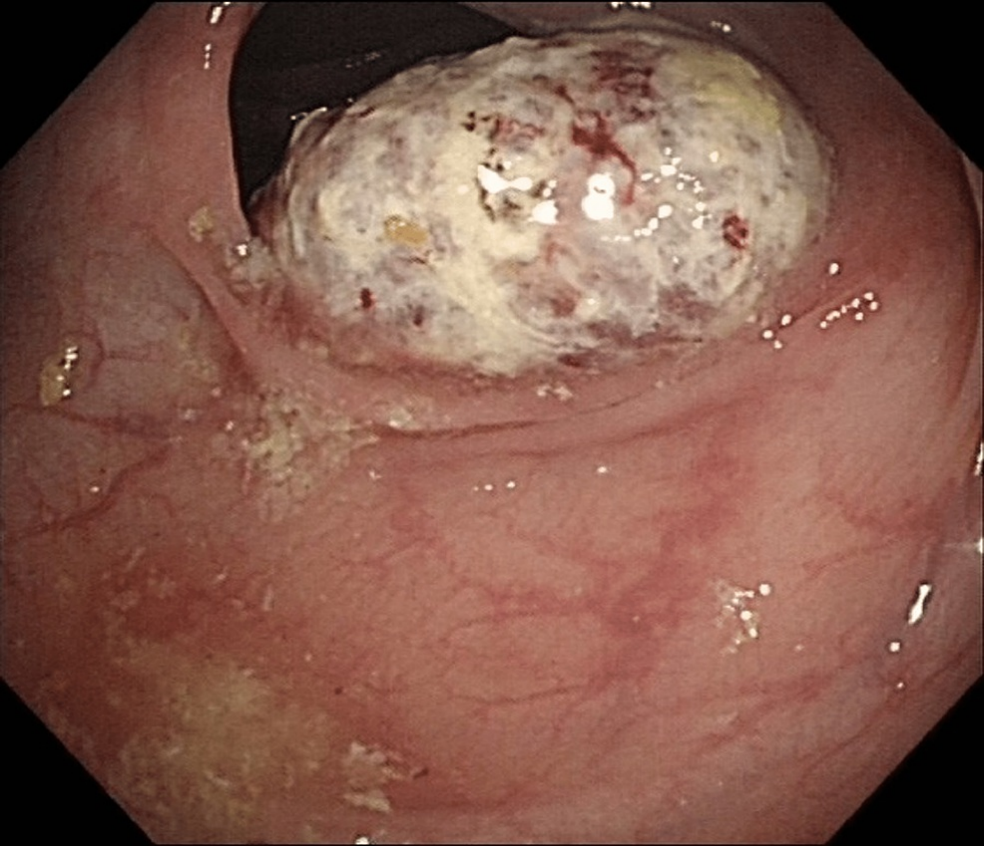
Endoscopic image of the ascending colonic lesion demonstrating ulceration and fibrin deposition.

**Figure 3:**
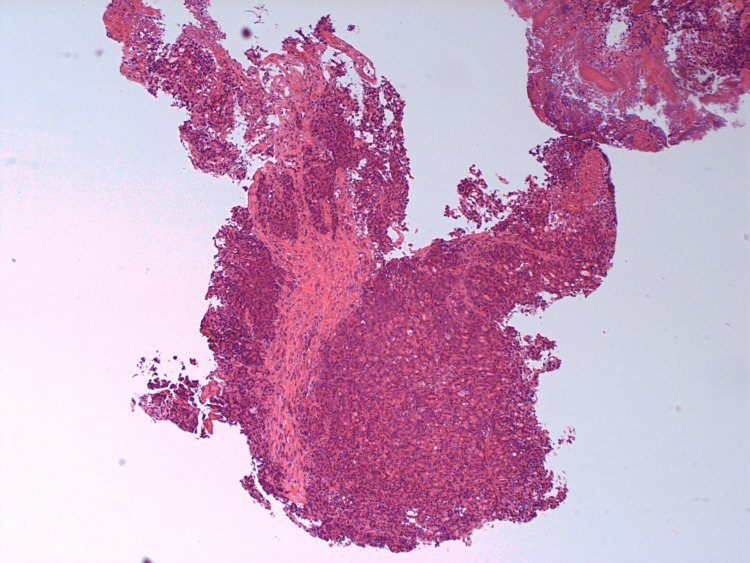
HA1 - H&E stains of the initial biopsy showing fragments of a poorly differentiated adenocarcinoma.

**Figure 4:**
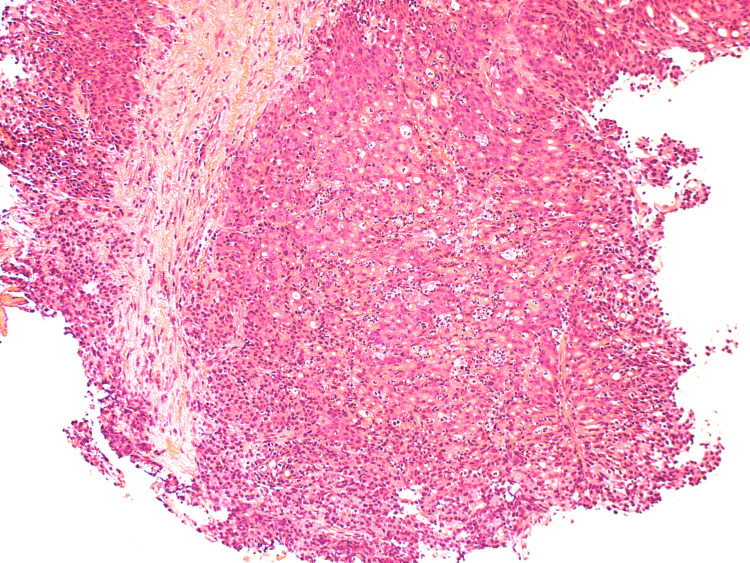
HA2 - H&E stains of the initial biopsy showing fragments of a poorly differentiated adenocarcinoma.

**Figure 5:**
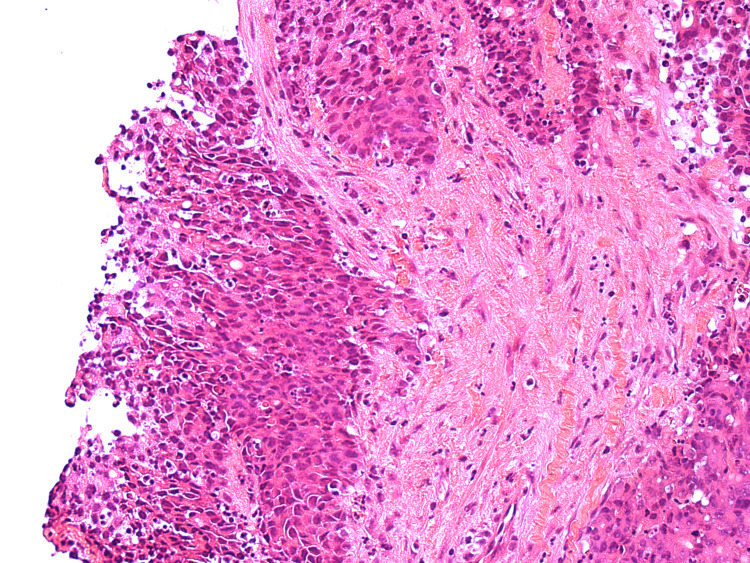
HA3 - H&E stains of the initial biopsy showing fragments of a poorly differentiated adenocarcinoma.

**Figure 6:**
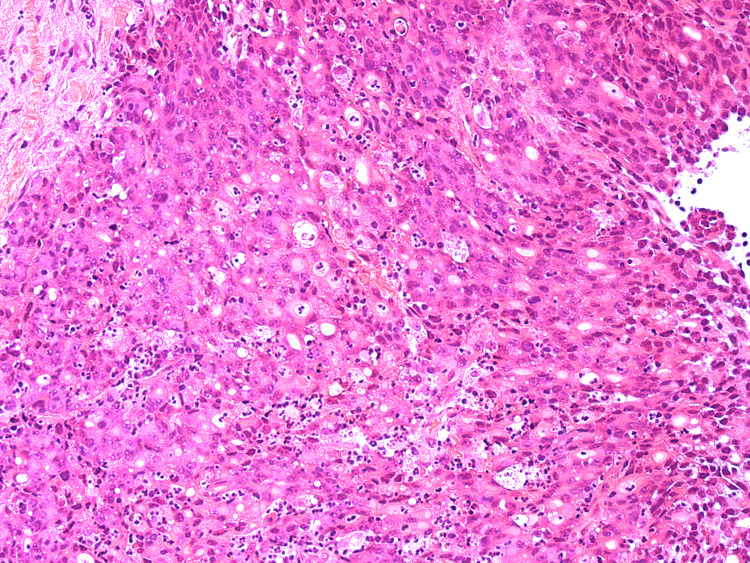
HA4 - H&E stains of the initial biopsy showing fragments of a poorly differentiated adenocarcinoma.

**Figure 7:**
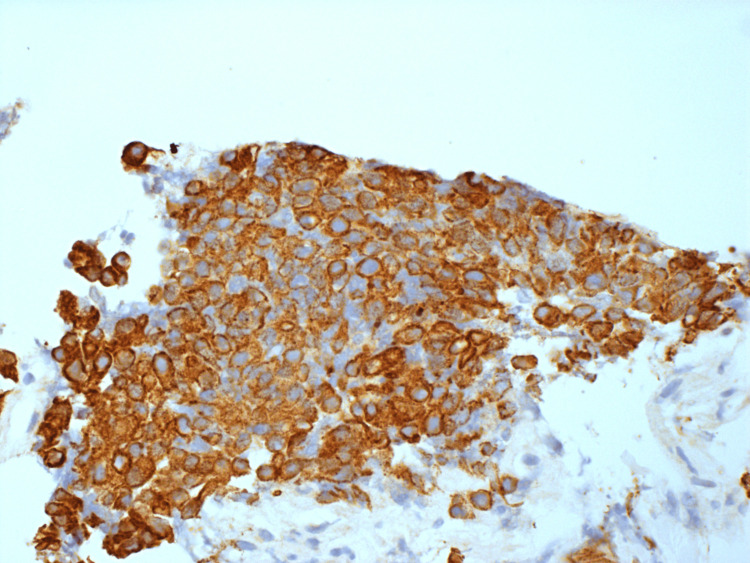
HA5 - immunostaining for cytokeratins (AE1/AE3) showing strong positivity in the tumor cells.

**Figure 8:**
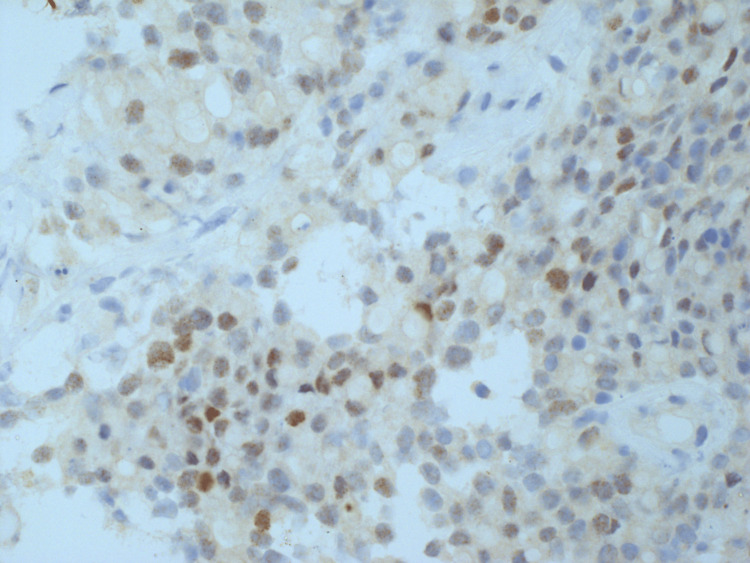
HA6 - immunostaining for CDX2 showing weak focal nuclear staining in the tumor cells.

A second attempted colonoscopy was abandoned due to uncontrolled hypertension mid-procedure. A third colonoscopy was completed successfully with cecal intubation confirmed. A 2.5 cm ulcerated lesion in the hepatic flexure which appeared to be malignant was identified and tattoos were placed distally at three places. Five biopsies were taken of the lesion. These suggested poorly differentiated carcinoma. Immunoprofile showed P40, LCA, CK7, and CK20-ve. GATA3 showed very weak positivity in background cells. CDX2 showed patchy nuclear positivity which was strong in places. This supported the likelihood of this being a colonic primary adenocarcinoma despite the lack of CK20 positivity (Figures 2-8).

MDT discussion was held and it was decided that she should have a right hemicolectomy as the evidence suggested she had a primary colonic adenocarcinoma. Pre-operative staging suggested that this was a T2/3N0M0 lesion. Other than the poorly controlled hypertension, there were no significant episodes of illness in the months prior to resection.

She underwent an uneventful laparoscopic right hemicolectomy in November 2018, five months post-initial referral, seven months post-onset of symptoms, and six weeks post-completed colonoscopy. The specimen included the tattooed area. There were no significant post-operative complications, and the patient was discharged on the sixth post-operative day.

**Figure 9:**
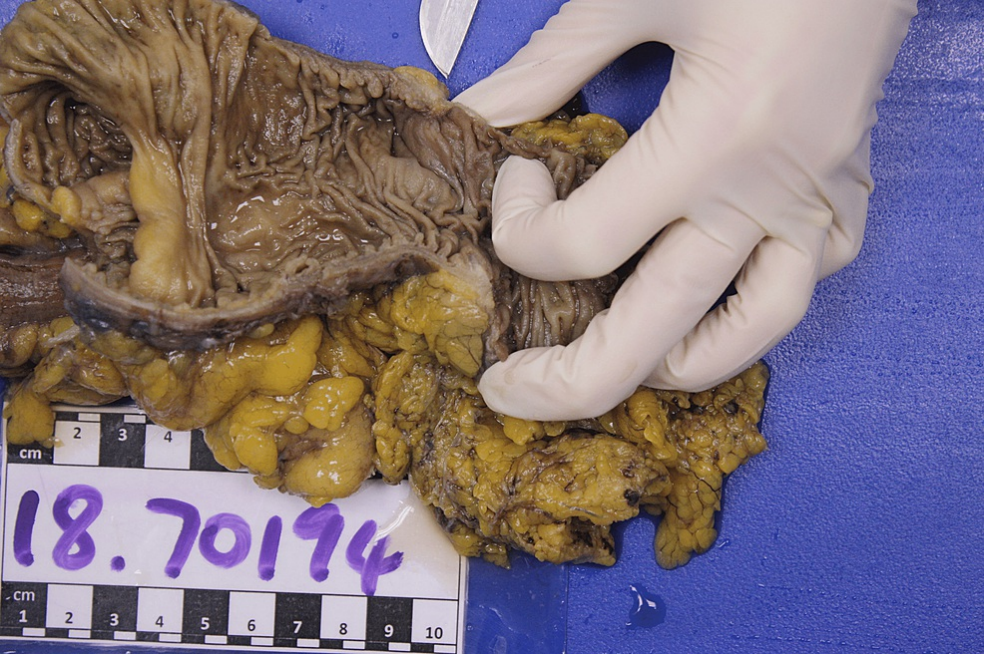
HA9 - gross photo of the resection specimen showing a small ulcerated stricture (pointed out with scalpel tip).

**Figure 10:**
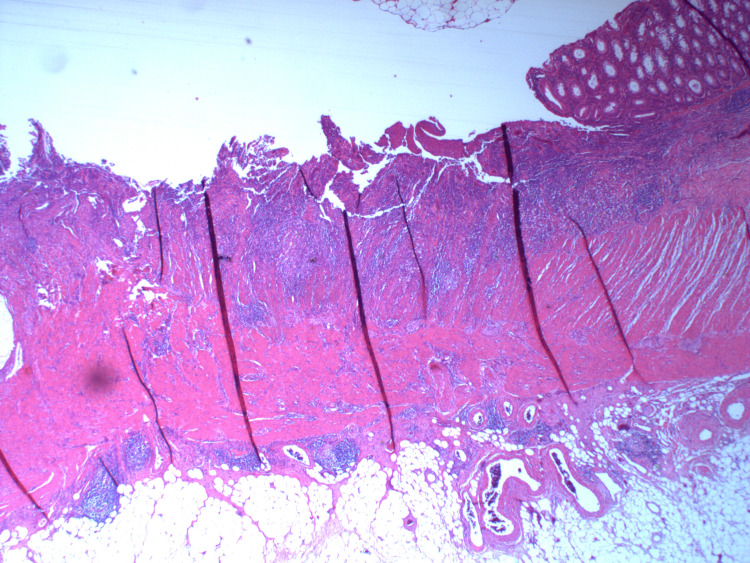
HA8 - H&E stains of sections from the bowel resection showing focal ulceration and an inflammatory infiltrate with no residual adenocarcinoma seen.

**Figure 11:**
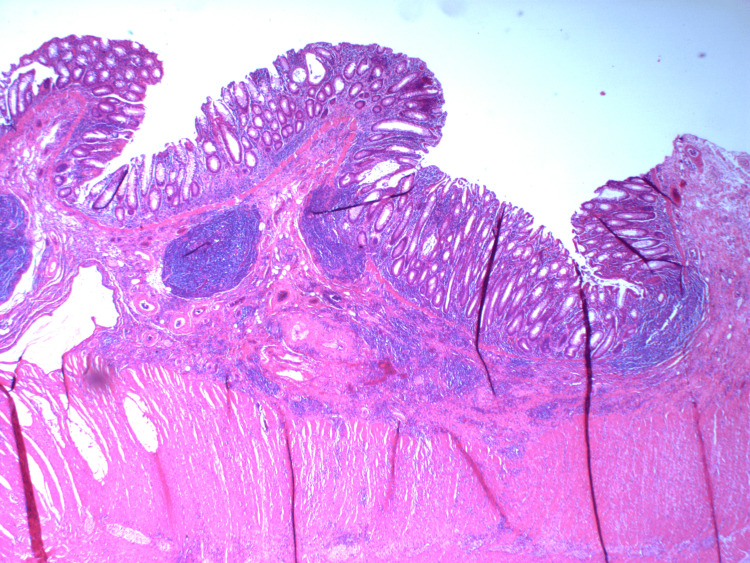
HA7 - H&E stains of sections from the bowel resection showing focal ulceration and an inflammatory infiltrate with no residual adenocarcinoma seen.

Subsequent histological examination of the specimen demonstrated that a specimen comprising 50 mm of terminal ileum and 125 mm of cecum and ascending colon. A small ulcerated lesion was seen measuring 8 mm in length and 6 mm in diameter, lying 110 mm from the proximal resection margin and 30 mm from the distal resection margin (Figure 9). Background mucosa was unremarkable. Histological examination of the ulcerated region showed a small focus on non-specific mucosal ulceration, with inflammatory reaction extending into the muscularis propria (Figures 10, 11). A few small mucin pools were noted within the muscularis propria at this point, but there was no evidence of dysplasia or invasive malignancy. Twenty-seven lymph nodes were identified which were negative. There was no evidence of malignancy within the resected specimen.

Further discussion at MDT and re-examination of the original biopsies confirmed the previous histological findings. The patient went on to have a colonoscopy with ileal intubation, with no evidence of an unresected tumor. A second opinion was sought on the preoperative biopsies and the resected specimen. Further histological examination confirmed the original findings of a poorly differentiated adenocarcinoma in the pre-operative biopsies, and an ulcerated, nodular area in the resected specimen, with no evidence of any residual cancer. We also reviewed the biopsies and colonoscopies of all procedures carried out that day, in case anything was mislabeled or there was a mix-up with identities. She was followed up as per Greater Manchester Cancer Network guidelines and there was no evidence of local or distant recurrence or metastasis.

Case 2

An 86-year-old female was referred to the surgical outpatient department in August 2020 on a two-week wait, urgent cancer referral pathway with change in bowel habits and loose bowel movements for several months. There was no history of weight loss, anemia, or iron deficiency. Her past medical history was limited, with only chronic lymphoid leukemia (CLL), stage A low-level lymphocytosis, with no requirement for treatment of significance. She was otherwise fit and well and did not take any significant medication.

**Figure 12:**
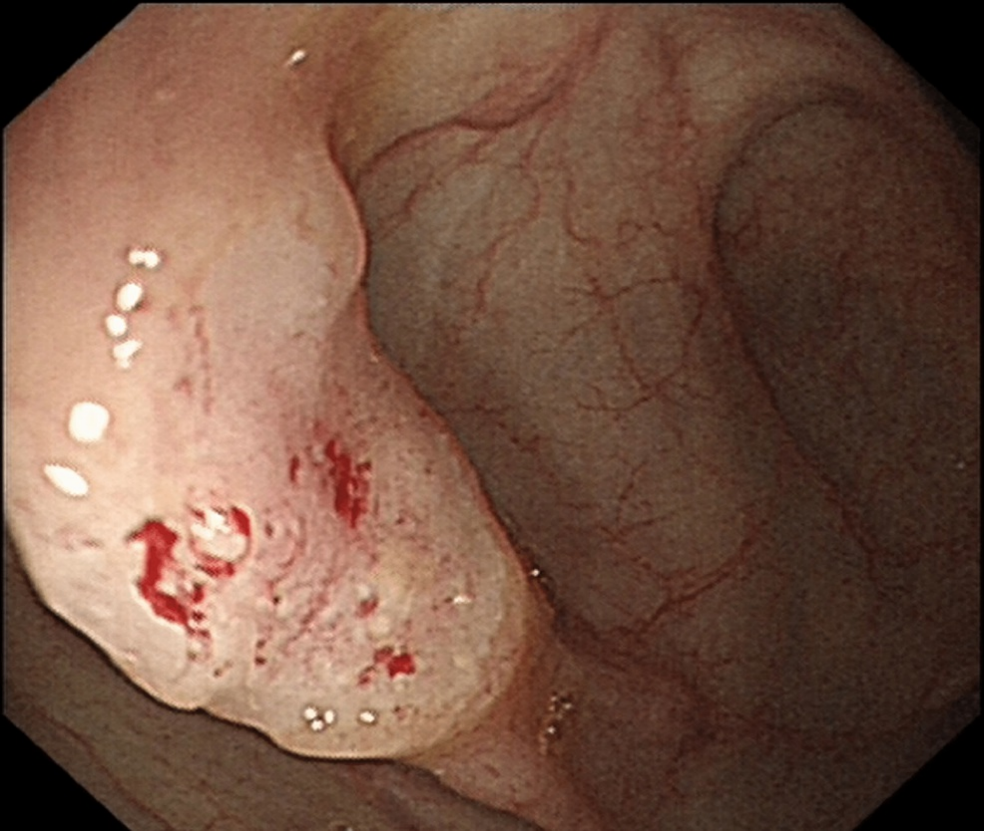
Endoscopic image showing malignant lesion.

**Figure 13:**
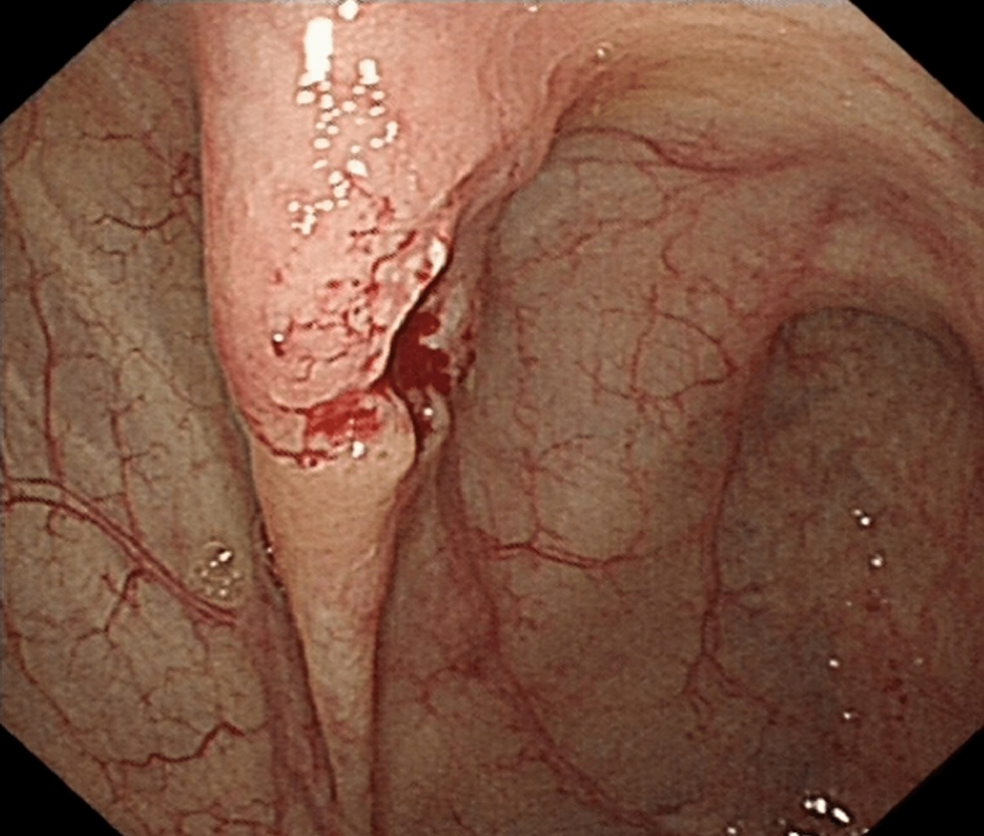
Lesion post-biopsy.

She underwent an elective straight-to-test colonoscopy, with no undue delay, which demonstrated a cecal lesion suspicious for a carcinoma. This was noted to be a sessile polypoidal lesion with Kudo type-5 pit pattern (Figure 12). Multiple biopsies were taken and a staging CT was arranged (Figure 13). As the lesion was cecal, no tattooing was performed. Subsequently, the patient was discussed in MDT.

Eight biopsies were taken consistent with a colonic adenocarcinoma with moderate differentiation. They showed colonic mucosa with a partly villous architecture, with high-grade dysplasia and areas of moderately differentiated adenocarcinoma.

It was suggested to be a possible T1 adenocarcinoma based on the CT imaging with the lesion being barely discernible on an unprepared CT. There were large right common iliac nodes and small left iliac and para-aortic nodes which were indeterminate radiologically and could have been indicative of nodal metastasis or related to her known diagnosis of CLL. MDT discussion suggested offering resectional curative surgery and to biopsy of the iliac nodes to determine their significance. After anesthetic assessment, she underwent elective laparoscopic right hemicolectomy and iliac node biopsy in September 2020 within seven weeks of referral and five weeks after her initial colonoscopy. There were no significant or unusual delays in her management. She had an uneventful post-operative recovery.

**Figure 14:**
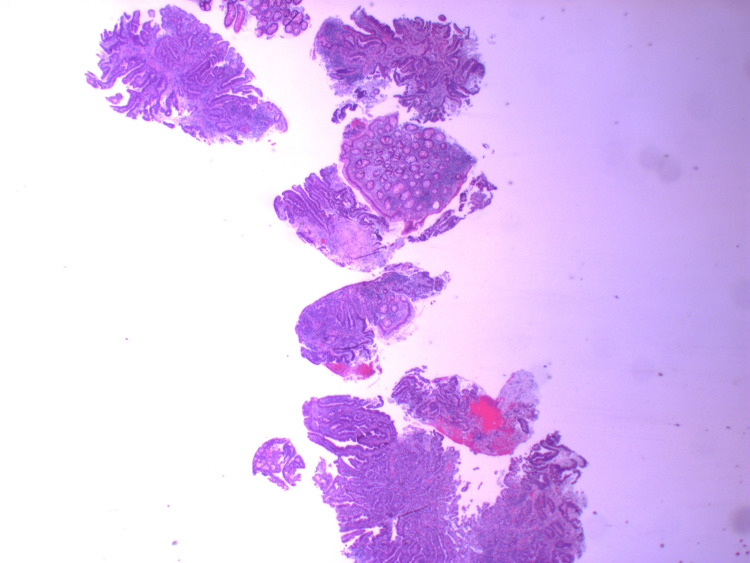
HA2.1 - H&E stains showing high-grade dysplasia and moderately differentiated adenocarcinoma.

**Figure 15:**
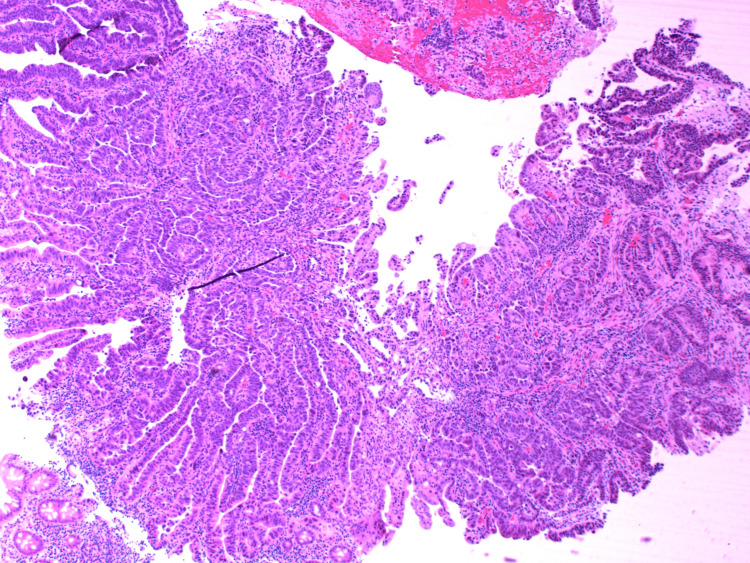
HA2.2 - H&E stains showing high-grade dysplasia and moderately differentiated adenocarcinoma.

**Figure 16:**
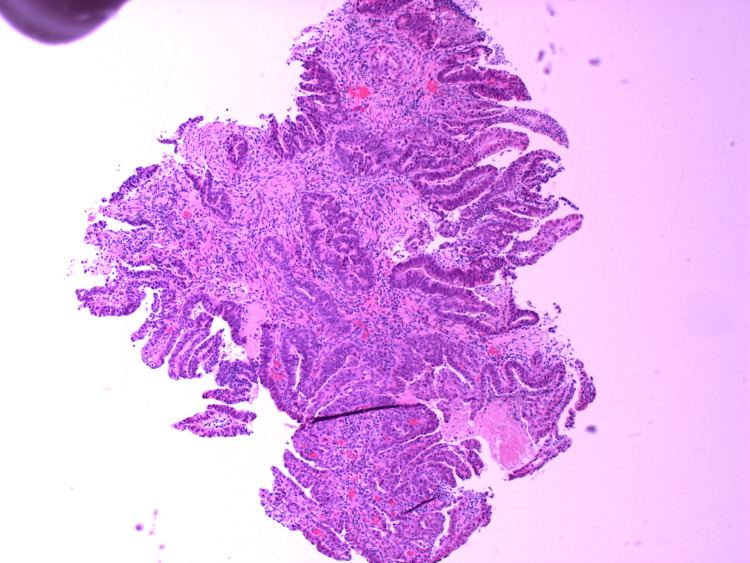
HA2.3 - H&E stains showing high-grade dysplasia and moderately differentiated adenocarcinoma.

**Figure 17:**
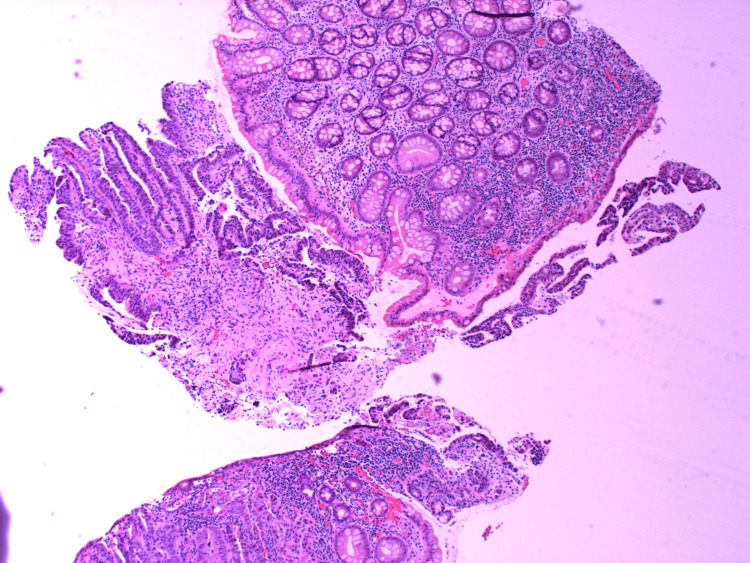
HA2.4 - H&E stains showing high-grade dysplasia and moderately differentiated adenocarcinoma.

**Figure 18:**
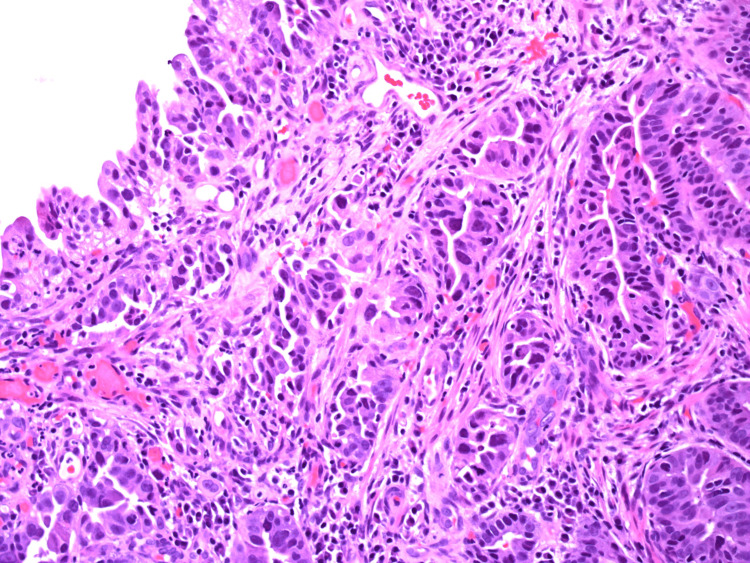
HA2.5 - H&E stains showing high-grade dysplasia and moderately differentiated adenocarcinoma.

Subsequent histological examination revealed no evidence of any significant mucosal lesion, tumor, or polyp. Thirteen lymph nodes were present, all negative for malignancy. The biopsied iliac node showed features consistent with CLL/small lymphocytic lymphoma. Colonic histology was reviewed by two histopathologists independently, and the excised lymph node was sent to the regional Hematological Oncology Diagnostic Service for a second opinion (Figures 14-18).

As in the first case, an investigation was carried out to exclude a mistake and a repeat colonoscopy was arranged. The colonoscopy was performed by two surgeons. There were no abnormal findings besides sigmoid diverticular disease and no evidence of cancer in any other segment of the colon. She has been followed up as per Greater Manchester Cancer Network Guidelines and remains cancer free at three and a half years.

## Discussion

The potential mechanisms of spontaneous regression are unclear, and several potential factors have been postulated. Historically Coley’s toxins were the first attempt at immunotherapy to promote regression, using injections of a heat-treated mixture of *Streptococcus pyogenes *and *Serratia marcescens *to stimulate a profound pyrexia [7]. This was initially thought to produce high levels of tumor necrosis factor (TNF) provoking an immune response but has more recently been suggested to be related to IL-12 levels [8]. The spontaneous regression of cancers has been linked to coincidental infections causing a response similar to Coley’s toxins. Abdelrazeq proposed many possible mechanisms including immunological, endocrine, metabolic, biopsy or debulking of the primary, elimination of a carcinogen or antigen (e.g., proximal diversion in bladder cancer), angiogenesis inhibition, tumor necrosis, oncogenes, growth factors, cytokines, genetic and epigenetic factors, induction of benign differentiation, apoptosis, and psychological factors [4]. Given the wide potential mechanisms, and the huge potential benefits should a harnessable mechanism be identified, it is important to report all potential cases. He identified several common factors pertaining particularly to patients with colorectal cancer - male predominance, a higher incidence in rectal cancer compared with colonic cancer, and a higher incidence in distal colonic tumors compared with proximal, a lack of occurrence in extra-peritoneal metastases.

There have been many proposed mechanisms for the spontaneous regression of cancer generally, and the true mechanisms are ambiguous. It is not clear in either of our cases what the potential mechanism behind the complete spontaneous regression could be. The first case had a significant delay between presentation and resection. The lesion showed significant fibrosis on post-operative histological examination, suggesting that an inflammatory event could have occurred during this delay period and that some form of immunological response could have been involved. On questioning, the patient did not recall any significant illnesses during the five-month period between diagnosis and resection. Cole postulated that trauma to the primary could stimulate an immune response, and it could be that the act of biopsying the lesion was of itself sufficiently traumatic to provoke inflammation [9]. The tumor was already showing significant signs of inflammation with slough and discoloration and was not typical for a colonic polyp (Figure 2).

Case 2 is a little different in so far as this was a smaller, sessile lesion, barely perceivable on CT, and relatively superficial. Eight biopsies were taken, and a review of the post-biopsy image demonstrates that these were fairly substantial, although there still appears to be some abnormal tissue on the endoscopic pictures after the biopsies have been taken (Figure 13). Again, this local trauma could have stimulated inflammation and an immune response, or it could be that the biopsies removed the only focus of cancer. The lack of any residual adenomatous tissue in the resected specimen would suggest that this is possible. It could be that the large deep biopsies caused a local reaction, and any residual adenomatous tissue underwent apoptosis. Adenomatous tissue was found in all the biopsies, and malignancy was found in more than two-thirds, and while it is not very likely, it is possible that the cancer was removed entirely. Unlike the first case, there was no mucosal lesion identified with no evidence of fibrosis in the resected specimen, suggesting that there are possibly different mechanisms. Our cases are unusual as they are both proximal cancers, and both female patients. Neither of these is common in colorectal cancers undergoing complete spontaneous regression.

The true rate of regression in colon cancer is not known for several reasons. Colon cancer is usually treated soon after diagnosis, and there is not usually a long delay with cancer frequently treated as an emergency. Current recommendations are that treatment should commence within four weeks [10]. No observational study of colon cancer with a randomized period of delay in the treatment could be performed and would not be considered ethical. A Norwegian observational study comparing the cumulative incidence of breast cancer between a screened and an otherwise comparable non-screened population over a six-year period found that the incidence in the non-screened group never reached the incidence in the screened group. They surmised that the missing cancers in the non-screened group either lay dormant or regressed without detection [11]. There will also be small cancers that never declare themselves clinically and have therefore developed and regressed entirely without the patient being aware. Any incidence of regression quoted can only ever be truly quoting the incidence of identified cancers and not the ones that go unidentified. Outcomes after delays brought about by the COVID pandemic may demonstrate an increased number of cases given the unusual delays in treatment. A systematic review of reported cases performed in a couple of years' time may demonstrate this and would be of interest.

There is evidence that spontaneous regression is more common in colorectal cancer displaying microsatellite instability and that these tumors are inherently more immunogenic [12,13]. Jessy discusses this paradox in a 2011 review, discussing how currently most drug treatments for cancer act more to suppress the immune system, thus opposing nature’s own defense mechanisms [14]. Immunotherapy is in its infancy and developments look tremendously promising and exciting as we enter a new age in cancer therapy.

## Conclusions

The spontaneous regression of cancer is well recognized, but the natural history of bowel cancer is not fully known and true rate of spontaneous regression within this cancer type is unclear. There are many things about the natural history of cancers we do not understand, and this will become more important as newer treatments and immune-based therapies become more prevalent. This study adds to the evidence of spontaneously resolving cancers and it is important to build up a picture of how often this occurs.
